# HLA-B and HLA-C Differ in Their Nanoscale Organization at Cell Surfaces

**DOI:** 10.3389/fimmu.2019.00061

**Published:** 2019-01-29

**Authors:** Philippa R. Kennedy, Charlotte Barthen, David J. Williamson, Daniel M. Davis

**Affiliations:** ^1^Faculty of Biology, Medicine and Health, Manchester Collaborative Centre for Inflammation Research, University of Manchester, Manchester, United Kingdom; ^2^Division of Cell and Molecular Biology, Imperial College London, London, United Kingdom

**Keywords:** HLA class I, membrane, nanoscale, super-resolution, HLA-B, HLA-C, immune synapse, NK cell

## Abstract

The particular HLA class I variants an individual carries influences their resistance and susceptibility to a multitude of diseases. Expression level and variation in the peptide binding region correlates with, for example, a person's progression to AIDS after HIV infection. One factor which has not yet been addressed is whether or not different HLA class I proteins organize differently in the cell membrane on a nanoscale. Here, we examined the organization of three HLA-B allotypes (B^*^2705, B^*^5301, and B^*^5701) and two HLA-C allotypes (C^*^0602 and C^*^0702) in the membrane of 721.221 cells which otherwise lack expression of HLA-B or HLA-C. All these allotypes are ligands for the T cell receptor and leukocyte immunoglobulin-like receptors, but additionally, the HLA-B allotypes are ligands for the killer-cell immunoglobulin-like receptor family member KIR3DL1, HLA-C^*^0602 is a ligand for KIR2DL1, and HLA-C^*^0702 is a ligand for KIR2DL2/3. Using super-resolution microscopy, we found that both HLA-B and HLA-C formed more clusters and a greater proportion of HLA contributed to clusters, when expressed at lower levels. Thus, HLA class I organization is a covariate in genetic association studies of HLA class I expression level with disease progression. Surprisingly, we also found that HLA-C was more clustered than HLA-B when expression level was controlled. HLA-C consistently formed larger and more numerous clusters than HLA-B and a greater proportion of HLA-C contributed to clusters than for HLA-B. We also found that the organization of HLA class I proteins varied with cell type. T cells exhibited a particularly clustered organization of HLA class I while B cells expressed a more uniform distribution. In summary, HLA class I variants are organized differently in the cell surface membrane which may impact their functions.

## Introduction

HLA class I (HLA-I) proteins are a critical component of the human immune response (1–4). Classical HLA-I present peptides to the T cell receptor (TCR) on CD8^+^ T cells, but innate cells are also capable of recognizing HLA-I using activating and inhibitory receptors, such as killer-cell immunoglobulin-like receptors (KIR) and leukocyte immunoglobulin-like receptors (LILR). It has been proposed that HLA diversity, which includes a high frequency of non-synonymous mutations in the peptide-binding region compared to elsewhere in the genome, is driven by selection pressure from pathogens that provide peptides to CTL (5, 6). KIR and LILR are also highly polymorphic, suggesting they too are under selection pressure for particular characteristics in their interactions with HLA-I (7, 8).

The importance of HLA-I in disease progression is best established in genetic studies. Variations in HLA-I, for example, correlate with the rate of progression to AIDS (9). If HIV-1 must mutate conserved epitopes to escape presentation by HLA-I, as is the case for HLA-B^*^2705/B^*^57, this is associated with a slower progression to AIDS (10, 11). Genetic associations between HIV-1 and HLA-I also implicate innate cells in controlling disease. Mutations of HIV epitopes that are fixed in individuals of a particular genetic background and enhance binding of HLA-I to inhibitory KIR (12, 13) or LILR (14) suggest that HIV is under selection pressure to inhibit innate immune responses as well as adaptive ones. Overall, it is still incompletely understood how particular HLA-I genes impact disease susceptibilities. One factor that has not yet been considered is whether or not variants of HLA-I organize differently in cell surface membranes.

## Results

### The Nanoscale Organization of Different HLA-I Allotypes

**Figure 1:**
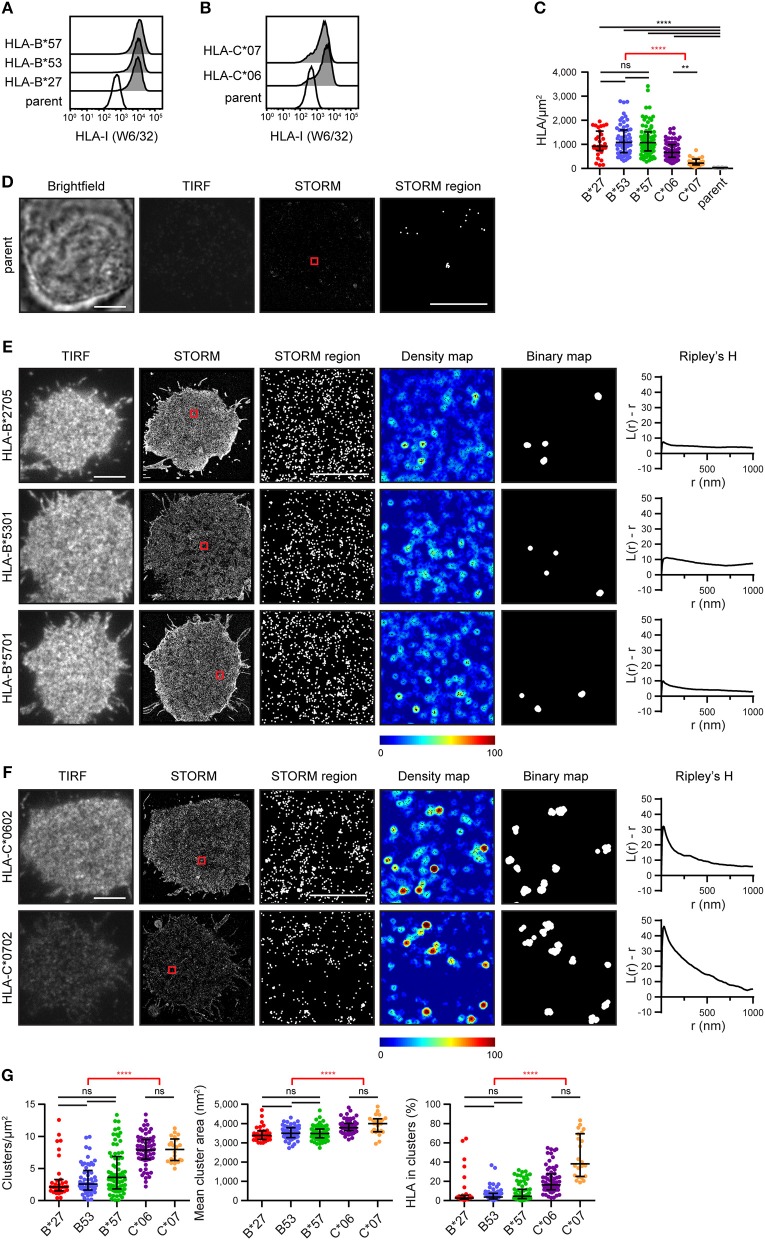
The nanoscale organization of different HLA-I allotypes. **(A,B)** Representative flow cytometry plots of 721.221 cells untransfected (“parent”; white histogram) or transfected to express **(A)** HLA-B or **(B)** HLA-C allotypes (gray histograms) stained for folded HLA-I. **(C)** The density of HLA-I in the membrane of transfected 721.221 cells when imaged by STORM. **(D)** Representative brightfield, TIRF microscopy and STORM images of 721.221 parent cells (scale bar: 5 μm). STORM panels show the Gaussian-rendered image of co-ordinate data. The 1 × 1 μm region (red box in STORM image) is enlarged and the corresponding scatter plot is displayed (STORM region; scale bar: 500 nm). **(E)** Representative TIRF microscopy and STORM images are shown for **(E)** HLA-B or **(F)** HLA-C allotypes (scale bar: 5 μm). STORM panels show the Gaussian-rendered image of co-ordinate data. The 1 × 1 μm regions (red boxes in STORM images) are enlarged and the corresponding scatter plots, density maps (G&F analysis) and binary maps are shown (scale bar: 500 nm). The Ripley's H function is plotted for that cell. **(G)** Quantitative analysis of nanoclusters. Each dot represents the mean value for a cell. Black bars show median and interquartile range. HLA-B^*^27 (red; 31 cells; 5 experiments); HLA-B^*^53 (blue; 58 cells; 10 experiments); HLA-B^*^57 (green; 71 cells; 11 experiments); HLA-C^*^06 (purple; 64 cells; 10 experiments); HLA-C^*^07 (orange; 22 cells; 5 experiments); parent (gray; 19 cells; 4 experiments). Kruskal-Wallis test with Dunn's multiple comparisons (black) and Mann Whitney test (for comparing all HLA-B v. HLA-C; red) (Graphpad Prism version7); ^**^p < 0.01, ^****^p < 0.0001.

A selection of *HLA-I* alleles (HLA-B^*^2705/^*^5301/^*^5701 and HLA-C^*^0602/^*^0702) were transfected into 721.221 cells that lack expression of classical HLA-I (15). Cells were sorted to express HLA-I at similar levels (Figures 1A,B). They were then plated on poly-L-lysine-coated glass, stained with mAb W6/32 that binds folded HLA-I and imaged by stochastic optical reconstruction microscopy (STORM). This technique provides co-ordinates for the location of HLA-I, with a resolution of < 20 nm (16). The density of HLA-I allotypes in the membrane was comparable, as detected by STORM, however C^*^0602 and C^*^0702 were at a lower density than the HLA-B allotypes (Figure 1C). As expected, almost no HLA-I were detected on untransfected 721.221 parental cells (Figures 1C,D).

Representative images of HLA-I on cells are shown for HLA-B (Figure 1E) and HLA-C (Figure 1F). The intensity of diffraction-limited total internal reflection fluorescence (TIRF) microscopy images (first column) reflects the relative density of HLA-I in the membrane of these cells (as evident in the STORM images; second column). In the enlarged regions of STORM images (third column), HLA-B exhibited a relatively homogenous distribution. This was confirmed by a Ripley's H analysis (final column) where the number of neighboring HLA-I was calculated for each detected HLA-I and adjusted for the expected number of neighbors in a random distribution. Positive values indicate clustering of HLA-I at the particular radius (r) examined. All three HLA-B allotypes had low peaks for Ripley's H values (< 20). These Ripley's H values were relatively similar across a number of radii, suggesting HLA-B was not clustering on any particular scale. In comparison, HLA-C allotypes had higher Ripley's H peaks (>20) that occurred at a radius of 30 nm, suggesting HLA-C was more clustered than HLA-B.

In order to make further comparisons between HLA-I organizations, we used custom software (17) to create maps of HLA-I clustering in the cell membrane (fourth column). HLA-I with an unexpectedly large number of neighbors are shown red whilst isolated HLA-I are blue. HLA-I above a clustering threshold form the basis of binary maps (fifth column), where clustered HLA-I are shown white. These binary maps allow quantitative comparison of HLA-I clusters (Figure 1G). When comparing different allotypes from the same gene, there were no significant differences in the density of clusters, mean cluster size or the proportion of HLA-I forming into clusters. A comparison of HLA-C with HLA-B revealed that HLA-C formed more clusters than HLA-B, these clusters were larger and a greater proportion of HLA-C contributed to clusters than for HLA-B. Therefore, in this case, where HLA-C was expressed at a lower level than HLA-B, HLA-C was consistently more clustered than HLA-B.

### Nanoscale Organization of HLA-B and -C Differs When Expression Is Controlled

**Figure 2:**
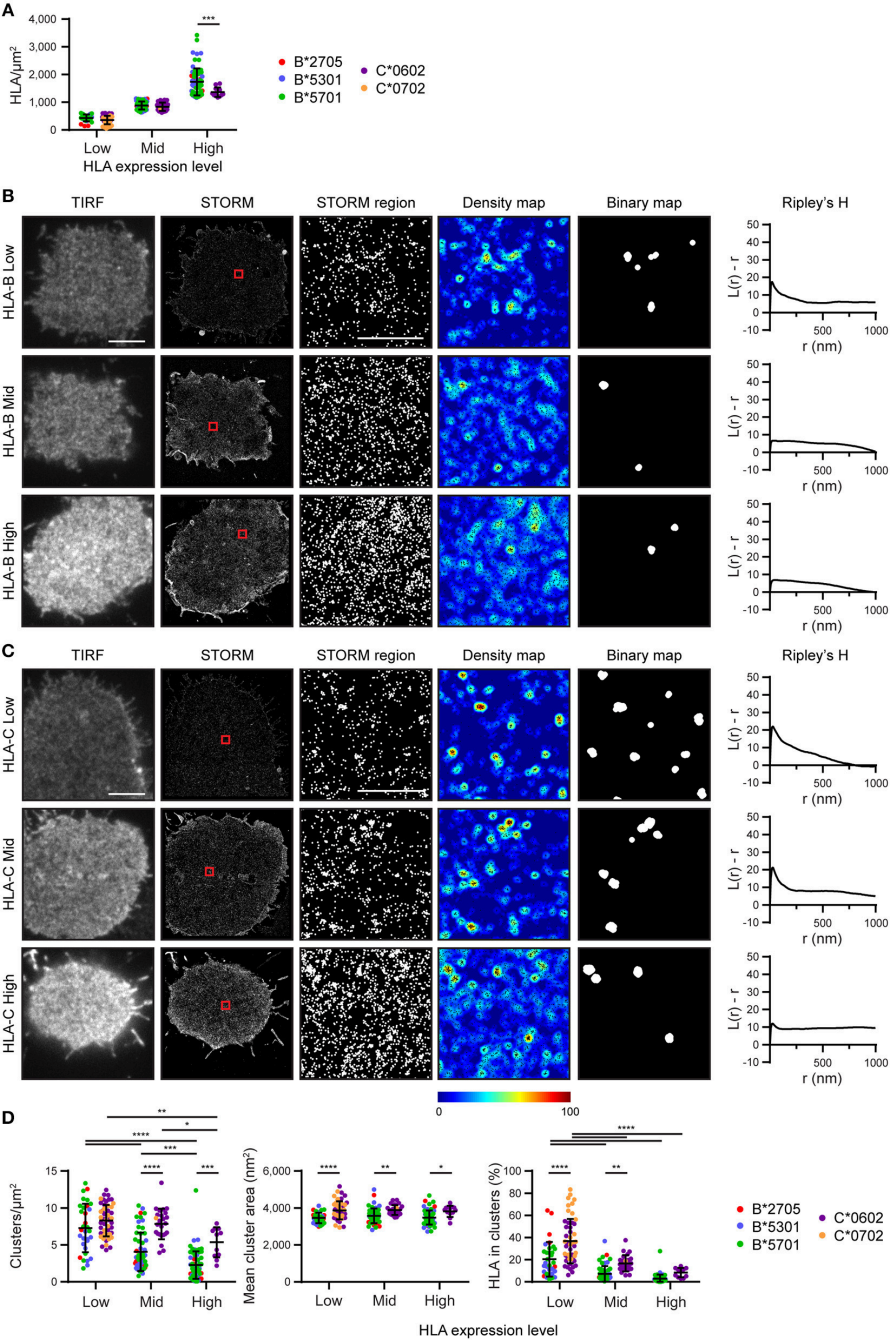
Nanoscale organization of HLA-I differs according to genes as well as expression level. HLA-I^+^ 721.221 cells analyzed in Figure 1 are presented stratified according to expression level. **(A)** The density of HLA-I in STORM images is plotted for the HLA-B or HLA-C allotypes within each group (Low, Mid, High expression). **(B,C)** Representative TIRF microscopy and STORM images are shown for **(B)** HLA-B or **(C)** HLA-C allotypes at different expression levels (scale bar: 5 μm). STORM panels show the Gaussian-rendered image of co-ordinate data. The 1 × 1 μm regions (red boxes in STORM images) are enlarged and the corresponding scatter plots, density maps (G&F analysis) and binary maps are shown (scale bar: 500 nm). The Ripley's H function is plotted for that cell. **(D)** Quantitative analysis of nanoclusters. Each dot represents the mean value for a cell and colors represent different allotypes. Black bars show mean and standard deviation. Two way ANOVA with Sidak's multiple comparison test (Graphpad Prism version7); ^*^p < 0.05, ^**^p < 0.01, ^***^p < 0.001, ^****^p < 0.0001.

We next set out to determine the effect of HLA-I density on nanoscale organization and to directly compare HLA-I allotype organization at different expression levels. To achieve this, STORM images were stratified into three groups according to the density of HLA-I (Figure 2A). Within these three groups (Low, Mid, High) there was no difference in HLA-I density between HLA-B and HLA-C in the low and mid groups although in the high group, HLA-B was at a higher density that HLA-C. It is therefore appropriate to focus on all three groups when considering the effect of expression level on HLA organization in general, but to focus on the low and mid-expressing groups for comparison between HLA-B and HLA-C.

Representative images of HLA-I on cells from the different tertiles are shown for HLA-B (Figure 2B) and HLA-C (Figure 2C). Again, HLA-B had lower peaks than HLA-C for Ripley's H values and these were relatively similar across a number of radii, suggesting HLA-B was not clustering on any particular scale. When considering the effects of expression level, cells with the lowest expression of HLA-I had the most clustered receptors, with higher Ripley's H peaks, more clusters and a greater proportion of HLA-I within clusters than the other two groups (Figure 2D). Higher density of HLA-B and HLA-C were associated with a more homogenous distribution of HLA-I and far fewer clusters. Cluster size was not affected by expression level. This is consistent with a model of HLA-I organization where a small proportion of HLA-I is clustered at low density, but that increased packing of HLA-I at higher density makes the organization more homogeneous and less clustered.

More surprisingly, when comparing HLA-B and HLA-C, HLA-C consistently formed larger clusters than HLA-B at each of the three expression levels examined. HLA-C also formed more clusters at the mid expression level than did HLA-B; and a greater proportion of HLA-C contributed to these clusters at both the low and mid expression levels. Thus, HLA-C is more clustered than HLA-B at the cell membrane, even when expression level is controlled.

### Nanoscale Clustering of HLA-C Is Not Determined by Cysteine at Residue 321

**Figure 3:**
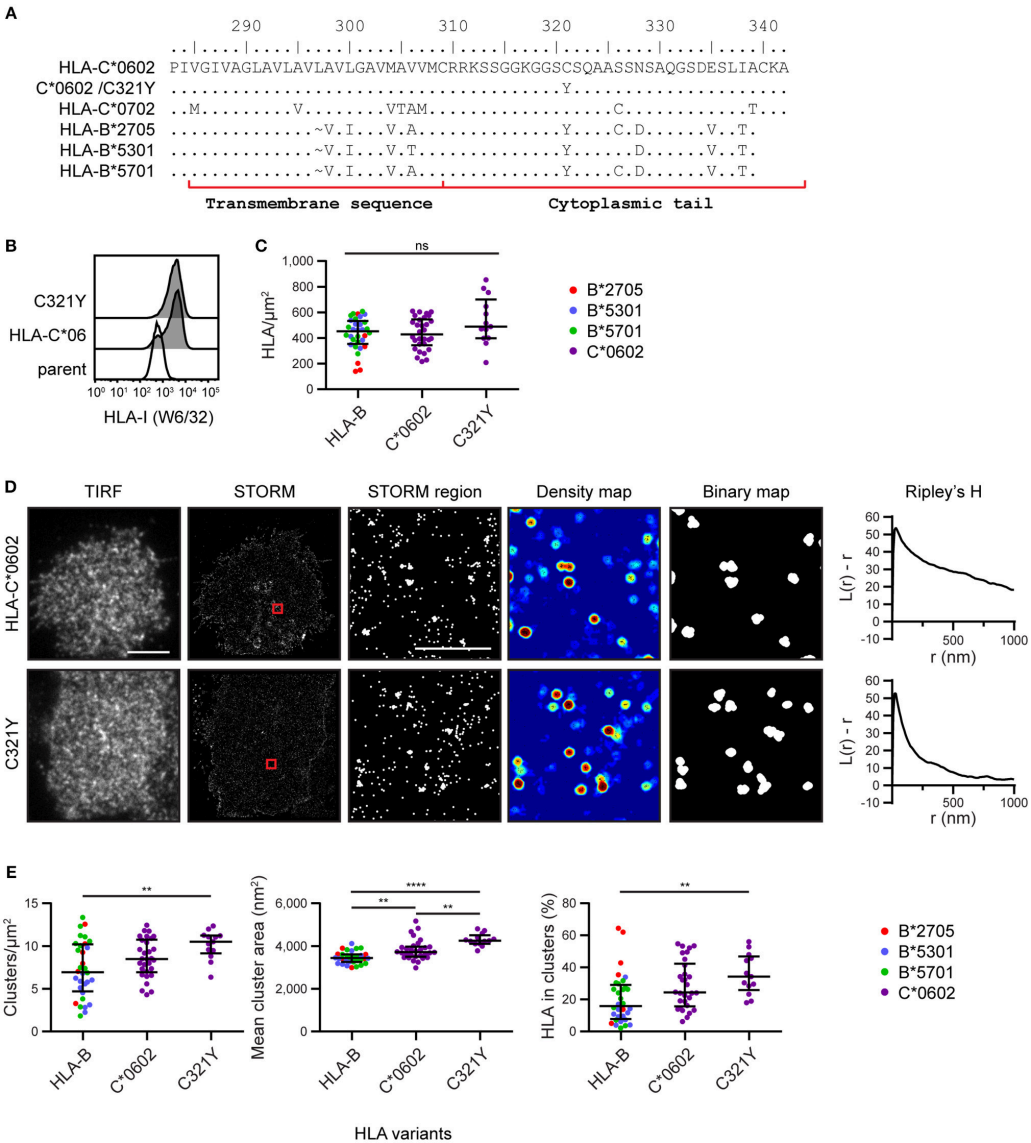
Nanoscale clustering of HLA-C is not determined by cysteine 321. **(A)** An alignment of amino acids in the C-terminus of HLA-I allotypes. The HLA-C allotypes examined here have a cysteine at position 321, while HLA-B allotypes have tyrosine. A point mutation was introduced to create HLA-C^*^0602/C321Y. **(B)** Representative flow cytometry plots of 721.221 cells untransfected (“parent”; white histogram) or transfected to express HLA-C^*^0602 or HLA-C^*^0602/C321Y (gray histograms) stained for folded HLA-I. **(C)** The density of HLA-I is plotted for HLA-C^*^0602/C321Y. The low expressing HLA-B allotypes and HLA-C^*^0602 (as shown in Figure 2) are plotted for comparison. **(D)** Representative TIRF microscopy and STORM images are shown for HLA-C^*^0602 and HLA-C^*^0602/C321Y (scale bar: 5 μm). STORM panels show the Gaussian-rendered image of co-ordinate data. The 1 × 1 μm regions (red boxes in STORM images) are enlarged and the corresponding scatter plots, density maps (G&F analysis) and binary maps are shown (scale bar: 500 nm). The Ripley's H function is plotted for that cell. **(E)** Quantitative analysis of nanoclusters. Each dot represents the mean value for a cell and colors represent different allotypes. Black bars show median and interquartile range. HLA-C^*^0602/C321Y (13 cells; 3 experiments); HLA-B (32 cells) and HLA-C (30 cells) as shown in Figure 2 low expressing group. Kruskal-Wallis test with Dunn's multiple comparisons (Graphpad Prism version7); ^**^p < 0.01, ^****^p < 0.0001.

To explore the mechanism by which HLA-C clusters more than HLA-B, we aligned the amino acid sequences of the HLA-I allotypes (18) used here for imaging (Figure 3A). We focused on the transmembrane sequence and cytoplasmic tail and identified residue 321 as a promising candidate to influence nanoscale organization. Residue 321 is Cys in HLA-C^*^0602/^*^0702 and Tyr in HLA-B^*^2705/5301/5701. To test whether this residue might control the clustering behavior that differentiates HLA-C from HLA-B, we introduced a point mutation into HLA-C^*^0602 to create a mutant with Tyr at this position (C321Y) and expressed this construct in 721.221 cells (Figure 3B). The density of HLA-C^*^0602 /C321Y was equivalent to the density of the low-expressing tertile for HLA-B and HLA-C (Figure 3C). A comparison of TIRF and STORM images (Figure 3D) revealed that the nanoscale organizations of HLA-C^*^0602 and HLA-C^*^0602 /C321Y were comparable. There was a similar density of clusters for these two variants (Figure 3E) and a similar proportion of HLA-C contributed to these clusters. HLA-C^*^0602 /C321Y still formed more clusters than HLA-B, which were larger and a greater proportion of HLA-I contributed to those clusters. Thus, this cysteine is not responsible for different clustering of HLA-C and HLA-B and the mechanisms by which HLA-C and –B cluster differently remain to be established.

### HLA-I on the Surface on B Cells Is More Homogeneous Than on Other Lymphocytes

**Figure 4:**
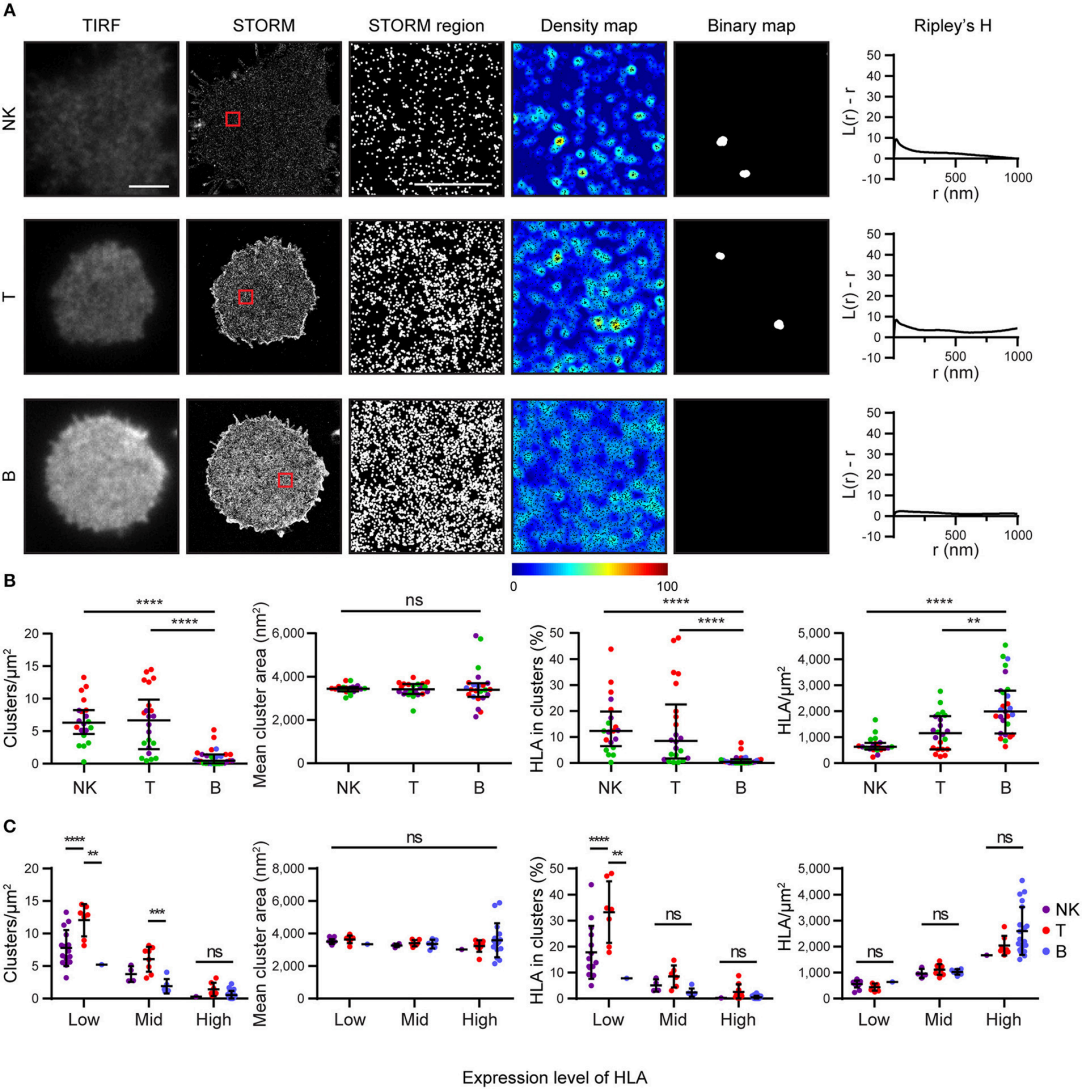
HLA on the surface on B cells is more homogeneous than on other lymphocytes. Lymphocytes isolated from peripheral blood by negative selection were stained for microscopy with mAb W6/32 that binds folded HLA-I. **(A)** Representative TIRF microscopy and STORM images of NK cells, T cells and B cells (scale bar: 3 μm). STORM panels show the Gaussian-rendered image of co-ordinate data. The 1 × 1 μm regions (red boxes in STORM images) are enlarged and the corresponding scatter plots, density maps (G&F analysis) and binary maps are shown (scale bar: 500 nm). The Ripley's H function is plotted for that cell. **(B)** Quantitative analysis of nanoclusters. Each dot represents the mean value for a cell and colors represent cells derived from different donors. Kruskal-Wallis test with Dunn's multiple comparisons. Black bars show median and interquartile range. **(C)** Lymphocytes were stratified into three groups (Low, Mid, High) according to the density of HLA-I in STORM images. Colors represent different cell types. Two way ANOVA with Tukey's multiple comparisons test. Black bars show mean and standard deviation. NK (20 cells; 3 donors); T (22 cells; 3 donors); B (27 cells; 4 donors) (Graphpad Prism version7); ^**^p < 0.01, ^***^p < 0.001, ^****^p < 0.0001.

We next examined how HLA-I was organized on the surface of primary human cells. We used mAb W6/32 to compare levels of folded HLA-I on NK, T and B cells. These cells were isolated by negative magnetic bead selection, plated on poly-L-lysine coated glass and imaged by STORM (Figure 4). We found that primary cells exhibit an innate variation of expression of HLA-I protein: NK cells and T cells had less HLA-I in the membrane than B cells (Figures 4A,B). NK cells and T cells exhibited more clusters with a greater proportion of HLA-I contributing to clusters than in B cells. At least some of this variation in HLA-I organization is due to different expression levels of HLA-I between cell types.

When cells were stratified according to HLA-I expression, it was apparent that T cells had a distinctive HLA-I organization compared to B cells and NK cells (Figure 4C). T cells exhibited more clusters than B cells or NK cells when HLA-I was expressed at low levels. The proportion of HLA-I contributing to clusters was also higher for T cells at low expression levels than for B or NK cells. There was no difference in HLA-I organization at high expression levels however. Cluster size did not vary, irrespective of expression level. These data show that HLA-I is organized differently on the surface of primary cells with B cells having a more homogeneous organization, NK cells having a more clustered organization, because of low HLA-I expression, and T cells having a more clustered organization of HLA-I even when expression level is accounted for.

## Discussion

Super-resolution microscopy has revealed that many immune cell surface receptors, membrane-proximal adaptors, and signaling molecules are constitutively organized in nanoscale structures (17, 19–24). However, one area that has not been explored so far, is whether or not genetic variability impacts this organization. The HLA system is the most polymorphic set of human genes which impacts our resistance and susceptibilities to different diseases (1–4). Thus, we set out to test whether or not HLA proteins encoded by different genes or alleles varied in their cell surface organization. We found that allotypes of the same locus did not differ in their nanoscale organization. However, between loci, a greater proportion of HLA-C assembled into clusters than for HLA-B. Moreover, HLA-C clusters were larger and more numerous at the surface, compared to HLA-B. This establishes that different HLA-I proteins, especially those encoded by different loci, exhibit distinct nanoscale organizations at cell surfaces.

721.221 cells can express low levels of non-classical HLA-E and -F which could feasibly impact our results. HLA-F is only a very minor fraction of HLA at the cell surface, evidenced by untransfected 721.221 having 10–40 fold less staining with W6/32 than any of the transfectants expressing HLA-B or -C. It remains to be established whether or not HLA-E impacts the overall organization of HLA by forming separate nanoclusters at the cell surface or by co-clustering with other HLA allotypes. It was not possible to compare HLA-C and HLA-B on primary cells, because each cell will express a combination of HLA and most HLA-I mAb cross-react. However, the difference between HLA-C and HLA-B is likely to be even more pronounced on primary cells, because HLA-C is far less expressed than HLA-B (25, 26). Levels of HLA-C expression are cell-type specific (27), so the different organization of HLA-I on T cells could reflect a different ratio of HLA-B and -C at the cell surface.

The mechanism by which HLA-I forms nanoscale clusters is unclear. Cysteine residues in the cytoplasmic tail have been shown to control HLA-B and HLA-C palmitoylation and stability (28), as well as their recognition by NK cells (29). We mutated HLA-C^*^0602 at Cys 321 to match HLA-B, but this failed to reduce the clustering of HLA-C relative to HLA-B. It remains to be seen if the cytoplasmic tails of HLA-I control their nanoscale organization and what other mechanisms could be involved. Membrane protrusions, for example, could be important in the formation of nanoscale HLA-I clusters, as has been shown for T cell receptor microclusters (30).

An implication of our results is that HLA class I organization is a covariate in genetic association studies which link HLA class I expression level with disease progression. In addition, levels of HLA expression vary widely in different organs and tissues, and upon local cytokine stimulation, which will also impact the protein's organization at cell surfaces. There is evidence that this will be functionally important; clustering of ligands has been shown to impact receptor triggering (31, 32). However, it is not easy to determine the impact of changes in HLA clustering because there is no simple way to alter the size, density, or spacing of clusters systematically and test the consequences. Being able to manipulate the mechanisms behind HLA clustering at cell surfaces, or nanoscale printing of HLA clusters, may be able to address this in the future. Clearly, a major new goal is to determine whether different organizations of HLA-I influences its recognition.

## Methods

### Cell Lines and Primary Cells

Blood was acquired from the NHS blood service under ethics license REC 05/Q0401/108 (University of Manchester). Lymphocyte populations were isolated and cultured as previously described, as were 721.221 cells (33) with the following additions: T cells were isolated using T cell isolation kit II (Miltenyi Biotec); NK cells were stimulated with 200 units/ml IL-2 (Roche), and all primary cells were rested at least 4 days prior to experiments.

### Generation of HLA-I-Expressing Cell Lines

Plasmid pMSCV expression vectors encoding HLA-B^*^2705/B^*^5301/B^*^5701 (34) were the kind gift of Malini Raghavan (University of Michigan, Michigan). These were cloned into pcDNA3.1 (ThermoFisher) using primers for HLA-B^*^27/B^*^53 (5′- TGATGAGGATCCATGCGGGTCACGGCG-3′; 5′-TGATGACTCGAGCTAAGCTGTGAGAGACACATCAGAGCC-3′) or HLA-B^*^57 (5′-TGATGAGGATCCATGCGGGTCACGGCA-3′; 5′-TGATGACTCGAGCTAAGCTGTGAGAGACACATCAGAGCC-3′; restriction sites underlined). These and pcDNA3.1 expression vectors containing HLA-C^*^0602 /C^*^0702 (35) or HLA-C^*^0602/C321Y (36) were transfected into 721.221 by electroporation. Stable cell lines obtained by limiting dilution were cultured in 1.6 mg/ml G418 (Sigma) to maintain expression.

### Flow Cytometry

Flow cytometry was performed as previously described (17) using W6/32 mAb.

### Microscopy

Cells were prepared in chambered coverglass (Lab-Tek, Nunc) pre-coated with 0.01% poly-L-lysine (Sigma-Aldrich). Cells were incubated on the glass for 20 min at 37°C, fixed in 4% formaldehyde for 20 min at room temperature (RT), then washed in PBS. Samples were then blocked for 1 h in 4% BSA/PBS and stained with AlexaFluor647-conjugated W6/32 mAb in 1% BSA/PBS for 1 h at RT. Samples were then washed in PBS, fixed again by 5 min incubation in 4% formaldehyde before a final wash. TIRF microscopy and STORM images were obtained with a wide-field SR GSD 3D microscope, as previously described (17). Forty thousand frames were acquired using 30% of the 647 nm laser power with samples in oxygen scavenging buffer (16).

### Image Processing

STORM images were processed as previously described (17). Poorly localized events were filtered to remove background (intensity <500; sigma>250; uncertainty >50 nm). Grid artifacts caused by saturated pixels were also removed (sigma = 160). Drift was corrected using cross-correlation and fluorophores across multiple frames were merged if localized within 35 nm and with up to 20 dark frames between them.

### Ripley's H Function Analysis

In the Ripley's K function analysis (37), each event is assigned a value, L(r), based upon the number of neighboring events within a given search radius (r). This value is adjusted in the Ripley's H function analysis, L(r)-r, to account for the increasing number of neighbors that would be expected in a random distribution as r increases. Therefore, a random distribution would have no peak on a Ripley's H function curve. STORM data derived from mAb labeled with several fluorescent moieties is inherently clustered, however, this analysis and the relative position of the Ripley's H function peak is informative for comparative study of receptors at the same density, because they are labeled to a similar extent.

### Clustering Analysis

Spatial point pattern analyses have been previously described (17) with Ripley's K function and univariate G&F analysis (38) used as the basis of quantitative clustering maps for STORM co-ordinate data. Clustering in density and binary maps was assessed for neighboring localisations within a 30 nm radius. Density maps were colored according to the L(30) value of individual localisations and the space between events colored by interpolation. HLA-I localisations with a Ripley's L(30) ≥70 were defined as clustered, since this threshold did not detect clusters in randomly distributed HLA-I at the same density as the images examined. To create binary maps, all clustered localisations were depicted by a white circle of radius 50 nm to create clusters which could be quantitatively compared.

## Data Availability Statement

The raw data supporting the conclusions of this manuscript will be made available by the authors, without undue reservation, to any qualified researcher.

## Author Contributions

PK performed experiments, supervised the project, analyzed data, and prepared the manuscript. CB conceived the project, performed experiments, and analyzed data. DW developed analysis methods. DD conceived and supervised the project and prepared the manuscript.

### Conflict of Interest Statement

The authors declare that the research was conducted in the absence of any commercial or financial relationships that could be construed as a potential conflict of interest.
